# Hepatitis B virus sero-profiles and genotypes in HIV-1 infected and uninfected injection and Non-injection drug users from coastal Kenya

**DOI:** 10.1186/s12879-015-1060-3

**Published:** 2015-07-30

**Authors:** Mark W. Kilongosi, Valentine Budambula, Raphael Lihana, Francis O. Musumba, Anthony K. Nyamache, Nancy L. M. Budambula, Aabid A. Ahmed, Collins Ouma, Tom Were

**Affiliations:** Department of Biomedical Sciences and Technology, Maseno University, Maseno, Kenya; Department of Environment and Health Sciences, Technical University of Mombasa, Mombasa, Kenya; Centre for Virus Research, Kenya Medical Research Institute, Nairobi, Kenya; Department of Microbiology, Kenyatta University, Nairobi, Kenya; Bomu Hospital, Mombasa, Kenya; Department of Biological Sciences, Embu University College, Embu, Kenya; African Population and Health Research Centre, Nairobi, Kenya; Department of Medical Laboratory Sciences, Masinde Muliro University of Science and Technology, P. O. Box 190–50100, Kakamega, Kenya

**Keywords:** Hepatitis B virus, HBV sero-markers, Sero-positivity, Genotypes, HIV-1, Injection drug users, Non-injection drug users, Coastal Kenya

## Abstract

**Background:**

Information about HBV sero-markers, infection stages and genotypes in HIV-1 infected and uninfected injection and non-injection drug users (IDUs) in Kenya remains elusive.

**Methods:**

A cross-sectional study examining HBV sero-marker, infection stages and genotypes was conducted among HIV-1 infected and uninfected, respectively, IDUs (*n* = 157 and *n* = 214) and non-IDUs (*n* = 139 and *n* = 48), and HIV-1 uninfected non-drug using controls (*n* = 194) from coastal, Kenya. HBV sero-marker and infection stages were based on HBV 5-panel rapid test plasma sero-reactivity. DNA was extracted from acute and chronic plasma samples and genotypes established by nested-PCR and direct sequencing.

**Results:**

HBsAg positivity was higher in HIV-1 infected IDUs (9.6 %) relative to HIV-1 uninfected IDUs (2.3 %), HIV-1 infected non-IDUs (3.6 %), HIV-1 uninfected non-IDUs (0.0 %) and non-drug users (2.6 %; *P* = 0.002). Contrastingly, HBsAb positivity was higher in HIV-1 uninfected IDUs (14.6 %) and non-IDUs (16.8) in comparison to HIV-1 infected IDUs (8.3 %), and non-IDUs (8.6 %), and non-drug users (8.2 %; *P* = 0.023). HBcAb positivity was higher in HIV-1 infected IDUs (10.2 %) compared to HIV-1 uninfected IDUs (3.3 %), HIV-1 infected non-IDUs (6.5 %), HIV-1 uninfected non-IDUs (2.1 %) and non-drug users (4.6 %; *P* = 0.038). Acute (5.7 %, 1.4 %, 0.0 %, 0.0 % and 1.5 %) and chronic (5.1 %, 0.9 %, 3.6 %, 0.0 % and 1.5 %) stages were higher in HIV-1 infected IDUs, compared to HIV-1 uninfected IDUs, HIV-1 infected and uninfected non-IDUs and non-drug users, respectively. However, vaccine type response stage was higher in HIV-1 uninfected IDUs (15.4 %) relative to HIV-1 infected IDUs (6.4 %), and HIV-1 infected (6.5 %), and uninfected (10.4 %) non-IDUs, and non-drug users (5.7 %; *P* = 0.003). Higher resolved infection rates were also recorded in HIV-1 uninfected IDUs (11.2 %) compared to HIV-1 infected IDUs (8.3 %), and HIV-1 infected (7.2 %), uninfected (6.3 %) non-IDUs, and non-drug users (6.7 %; *P* = 0.479), respectively. Only A1 genotype showing minimal diversity was detected among the study participants.

**Conclusion:**

HBV sero-markers and infection staging are valuable in diagnosis and genotyping of HBV infections. Among IDUs, higher HBsAg and HBcAb positivity in HIV-1 infected and higher HBsAb positivity in HIV-1 negative IDUs suggests frequent exposure. Additionally, HBV genotype A is the dominant circulating genotype in both high and low risk populations of Kenya.

## Background

Despite existence of an effective vaccine, about 2 billion people have been exposed to hepatitis B virus (HBV) leading to at least 300 million chronic HBV infections and 0.6 million deaths worldwide [1]. The problem is further compounded by the spreading co-endemicity with HIV-1 leading to at least three million co-infections in the world [2]. The higher rates of HBV and HIV-1 co-infections are driven by the shared portals of entry such as parenteral, perinatal and sexual routes [3].

Hepatitis B virus sero-prevalence is determined by examining for the presence of hepatitis B surface antigen (HBsAg), hepatitis B surface antibody (HBsAb), hepatitis B pre-core antigen (HBeAg), hepatitis B pre-core antibody (HBeAb) and hepatitis B core antibody (HBcAb) sero-marker reactivity [4]. HBsAg represent active acute or chronic infection, HBeAg indicate high viral replication and persistence while HBsAb and HBeAb signify hepatitis B resolution [4, 5]. HBcAb is a non-protective total antibody of the classes IgM and IgG denoting acute-window and past infection, respectively [4]. To our knowledge, no study has estimated HBV sero-prevalence by concurrent testing of the five hepatitis B virus sero-markers in Kenya.

The incubation period following HBV exposure varies from one to six months and correlates with magnitude of the inoculum [6]. Resolution and outcomes of HBV infection are governed by host immunity, age, gender, infection route and genotype [7]. While immune competent individuals successfully resolve HBV infection [8], immune-suppressed subjects such as HIV-1 infected patients rapidly progress from acute to chronic stages [9]. Although most individuals recover from acute or mild infections, occult infection persists in the liver with reactivation largely occurring in immune-suppressed individuals [10]. In Sub-Saharan Africa, HBV and HIV-1 are co-endemic [11], but information about HBV infection stages in HIV-1 infected and uninfected injection drug users (IDUs) and non-IDUs is unknown.

To date, ten genotypes (A-J) of HBV have been reported with Africa having mainly genotype A and to a varying extend genotypes C, D and E [12]. Although genotype A is the most common in blood donors, commercial sex workers and HIV-1 infected patients on antiretroviral treatment (ART) in Kenya, genotypes D and E occur less frequently [13–17]. HBV genotypes influence disease severity and response to antiviral treatment, and cluster with risk-population [10, 18]. However, circulating HBV genotypes in most-at-risk populations (MARP) in Kenya particularly among IDUs and non-IDUs remains elusive. This study, therefore, examined hepatitis B virus sero-markers, infection stages and genotype distribution in HIV-1 infected and uninfected IDUs and non-IDUs residing at Coastal Kenya.

## Methods

### Study area, design and population

A cross-sectional study of drug users was conducted at Bomu Hospital, Mombasa, coastal Kenya. A description of the study site and recruitment procedure is presented elsewhere [19]. Injection drug users were defined as individuals exhibiting needle-scars and reporting use of any illicit injection drug from the United Nations Office on Drugs and Crime (UNODC) report [20] for at least once in the previous month. Non-injection drug users were defined as persons who had never injected drugs but have used at least one non-injection drugs listed in the UNODC report [20]. The study participants were stratified into HIV-1 infected and uninfected IDUs, HIV-1 infected and uninfected non-IDUs, and HIV-1uninfected non-drug using controls (from the general population). About, 3.5 mL venipuncture blood was collected in EDTA BD vacutainer^TM^ anticoagulant tubes (BD, Franklin Lakes, USA) for HIV-1 serological testing, CD4+ T cell enumeration and plasma harvesting as described elsewhere [19].

### HBV sero-marker testing

The five HBV serological markers (HBsAg, HBsAb, HBeAg, HBeAb and HBcAb-IgM) reactivity of plasma samples was determined using the one-step HBV-5 panel rapid diagnostic cassette (Healthaw Medical limited, Hangzhou, China) according to the manufacturer’s instructions. Briefly, 5 μL of the test plasma was placed into each of the five sample wells corresponding to the sero-markers. Two drops of buffer were added to each well and HBV sero-marker reactivities recorded after 20 min.

### Sequencing and genotyping

Hepatitis B virus DNA was extracted from acute and chronic plasma samples using QiaAmp™ DNA Mini Kit (Qiagen Inc., Valencia, USA) according to the manufacturer’s recommendations. Hepatitis B virus *PreS1* gene was amplified by nested polymerase chain reaction (PCR) using GeneAmp™ PCR system 9700 (Applied Biosystems, Foster City, USA). Each PCR reaction contained 12.5 μL of 2x Phusion high-fidelity PCR master mixes, 1.25 μL of each primer, 5 μL of DNA and DEPC-treated water in a final volume of 25 μL. Amplification was performed using the primers HBPr1 (position: 2850–2868, 5'-GGGTCACCATATTCTTGGG-3') and HBr135 (position: 803–822, 5'- CAAAGACAAAAGAAAATTGG-3') for the first round, and HBPr2 (position: 2867–2888, 5'-GAACAAGAGCTACAGCATGGG-3') and HBPr3 (position: 3226–3246, 95'-CCACTGCATGG CCTGAGGATG-3') for the second round [21]. The first and second round PCR conditions were one cycle at 94 °C for 10 min, followed by 40 cycles at 94 °C for 30 s, 50 °C for 30 s, and 72 °C for 1 min, with a final extension of 72 °C for 10 min. PCR products were visualized by an agarose gel electrophoresis method. Purified DNA products were directly sequenced using automated DNA sequencer ABI 377 (Applied Biosystems, Foster City, USA), using fluorescence-labelled dideoxynucleotide chain terminators (ABI Prism BigDye™ Terminator Cycle Sequencing Reaction kit; Applied Biosystems). About 1.25 μL of primers HBPr2 and HBPr3 were used for forward and reverse sequencing reactions, respectively. Pairwise contiguous sequences were generated using DNA Baser Sequence Assembler version 4.20.0 (Heracle Software, Germany). Consensus sequences were aligned with complete HBV genotypes A-J reference sequences from Genbank using ClustalW [22]. Phylogenetic analyses was performed using MEGA version 6 [23]. 

### Statistical analysis

Statistical analyses were performed using SPSS version 19.0 (IBM SPSS Statistics for Windows, Version 19.0. Armonk, NY: IBM Corp.). Age and CD4+ T cell counts were compared among groups using Kruskal Wallis test followed by Dunn’s post-hoc correction for multiple comparisons. Differences in the distribution of proportions of gender, HBV sero-markers, infection stages and genotypes were compared across the study groups using the Chi-square tests. All tests were 2-tailed with statistical significance set at a critical alpha value of 5 %.

### Ethical considerations

The study was approved by Kenyatta University Ethical Review Committee and was conducted according to the Helsinki Declarations [24]. Written informed consent was obtained from each participant before enrolment and confidentiality was ensured in the course of the study. All the study participants were provided with free health education on sexually transmitted infections (STIs) including HIV, hepatitis B and C, tuberculosis, hygiene and nutrition. Participants testing positive for HIV were referred to the comprehensive care centres at Bomu Hospital or the Coast General Referral Hospital for treatment, care and support.

## Results

### Baseline characteristics

The baseline characteristics of the study participants are summarized in Table 1. A total of 752 adults were recruited into the study. The study participants were categorized into the following five study groups: 1) HIV-1 infected IDUs (*n* = 157); 2) HIV-1 uninfected IDUs (*n* = 214); 3) HIV-1 infected non-IDUs (*n* = 139); 4) HIV-1 uninfected non-IDUs (*n* = 48) and HIV-1 negative non-drug users (*n* = 194). HIV-1 uninfected IDUs (6.5 %) had fewer females compared to HIV-1 infected IDUs (54.8 %), HIV-1 infected non-IDUs (60.4 %), HIV-1 uninfected non-IDUs (39.6 %) and HIV-1 uninfected non-drug users (58.8 %; *P* < 0.0001). Age varied significantly across the study groups (*P* < 0.0001) with HIV-1 infected non-IDUs being older (median, 36.0; IQR, 14.5) than HIV-1 uninfected non-IDUs (median, 30.4; IQR, 11.8), and non-drug users (median, 30.8; IQR, 12.8; *P* < 0.001). CD4+ T cell counts were significantly different across the groups (*P* < 0.0001) such that HIV-1 infected IDUs (median, 456; IQR, 449) presented with lower counts relative to HIV-1 uninfected non-drug users (median, 831; IQR, 513), HIV-1 uninfected non-IDUs (median, 809; IQR, 486), HIV-1 infected non-IDUs (median, 553; IQR, 479) and HIV-1 uninfected IDUs (median, 905; IQR, 641; *P* < 0.001). In addition, HIV-1 infected non-IDUs (median, 553; IQR, 479) had lower CD4+ T cell counts compared to HIV-1 uninfected non-drug users (median, 831; IQR, 513), HIV-1 uninfected non-IDUs (median, 809; IQR, 486) and HIV-1 uninfected IDUs (median, 905; IQR, 641; *P* < 0.001).

**Table 1:** Baseline characteristics of the study participants

	Non-drug users	Non-injection drug users		Injection drug users		
*Characteristic*	*HIV-1(−), n = 194*	*HIV-1(−), n = 48*	*HIV-1(+), n = 139*	*HIV-1(−), n = 214*	*HIV-1(+), n = 157*	*P*
Females, n (%)	114 (58.8)	19 (39.6)	84 (60.4)	14 (6.5)	86 (54.8)	<0.0001
Age, yrs.	30.8 (12.8)	30.4 (11.8)	36.0 (14.5)^a,b^	31.7 (9.1)	30.6 (6.5)	<0.0001
CD4+ T cells/μL	831 (513)	809 (486)	553 (479)^a,b,c^	905 (641)	456 (449)^a,b,c^	<0.0001

Data shown are number (n) and proportions (%) of subjects for gender and medians (IQR, interquartile range) for age and CD4+ T cell counts. Statistical comparison of proportions among groups was conducted by Chi-Square test. Age and CD4+ T cell comparisons across groups were performed using Kruskal Wallis test followed by Dunn’s post-hoc test for multiple comparisons. HIV-1(+), human immunodeficiency virus-1 infected, HIV-1(−) uninfected. ^a^ *P* < 0.001 vs. non-drug users; ^b^ *P* < 0.001 vs. HIV-1(−) non-injection drug users; and ^c^ *P* < 0.001 vs. HIV-1(−) injection drug users

### Hepatitis B viral marker distribution

Hepatitis B sero-marker reactivities in the study participants are shown in Table 2. HBsAg positivity rates were significantly higher in HIV-1 infected IDUs (9.6 %) compared to HIV-1 uninfected IDUs (2.3 %), HIV-1 infected non-IDUs (3.6 %), HIV-1 uninfected non-IDUs (0.0 %) and HIV-1 uninfected non-drug users (2.6 %; *P* = 0.002). Interestingly, rates of HBsAb positivity were significantly higher in HIV-1 uninfected IDUs (16.8 %) and HIV-1 uninfected non-IDUs (14.6 %), in comparison to HIV-1 infected IDUs (8.3 %), HIV-1 infected non-IDUs (8.6 %) and HIV-1 uninfected non-drug users (8.2 %; *P* = 0.023). Although only 1 (0.13 %), HBeAg positive reaction was detected among the study groups, rates of HBeAb positivity were similar across study groups (*P* = 0.186). Consistent with the pattern observed for HBsAg reactivity, HBcAb-IgM positivities were higher in HIV-1 infected IDUs (10.2 %) compared to HIV-1 uninfected IDUs (3.3 %), HIV-1 infected non-IDUs (6.5 %), HIV-1 uninfected non-IDUs (2.1 %) and HIV-1 uninfected non-drug users (4.6 %; *P* = 0.038).

**Table 2:** Hepatitis B virus sero-reactivities, infection stages and genotypes in the study participants

	Non-drug users	Non-injection drug users		Injection drug users		
	*HIV-1(−), n = 194*	*HIV-1(−), n = 48*	*HIV-1(+), n = 139*	*HIV-1(−), n = 214*	*HIV-1(+), n = 157*	*P*
*Sero-marker*						
HBsAg	5 (2.6)	0 (0.0)	5 (3.6)	5 (2.3)	15 (9.6)	0.002
HBsAb	16 (8.2)	7 (14.6)	12 (8.6)	36 (16.8)	13 (8.3)	0.023
HBeAg	0 (0.0)	0 (0.0)	1 (0.7)	0 (0.0)	0 (0.0)	-
HBeAb	13 (6.7)	3 (6.3)	13 (9.4)	26 (12.1)	21 (13.4)	0.186
HBcAb-IgM	9 (4.6)	1 (2.1)	9 (6.5)	7 (3.3)	16 (10.2)	0.038
*Infection stage*						
1	3 (1.5)	0 (0.0)	0 (0.0)	3 (1.4)	9 (5.7)	-
2	3 (1.5)	0 (0.0)	5 (3.6)	2 (0.9)	8 (5.1)	-
3	11 (5.7)	5 (10.4)	9 (6.5)	33 (15.4)	10 (6.4)	0.003
4	13 (6.7)	3 (6.3)	10 (7.2)	24 (11.2)	13 (8.3)	0.479
5	164 (84.5)	40 (83.3)	115 (82.7)	152 (71.0)	117 (74.5)	0.005
*HBV genotypes*						
A1	6 (3.1)	0 (0.0)	3 (2.2)	4 (1.9)	10 (6.4)	-

Data shown are number (n) and proportions (%) of subjects. HIV-1(+), human immunodeficiency virus-1 infected, HIV-1(−) uninfected. HBsAg, hepatitis B surface antigen; HBsAb, hepatitis B surface antibody; HBeAg, hepatitis B e-antigen; HBeAb, hepatitis B e-antibody; HBcAb-IgM, hepatitis B core antibody Immunoglobulin M. Statistical comparison was performed using Pearson’s Chi-square test where appropriate. Hepatitis B sero-marker test results were used to categorize hepatitis B virus infection status in to five (1–5) infection stages based on previous classifications [4, 5, 42, 43] as follows: 1 = Acute (HBsAg+, HBsAb-, HBeAg+/−, HBeAb-, HBcAb-IgM+/−); 2 = Chronic (HBsAg+, HBsAb+/−, HBeAg+/−, HBeAb+, HBcAb-IgM+/−); 3 = Vaccine type response (HBsAg-, HBsAb+, HBeAg-, HBeAb-, HBcAb-IgM-); 4 = Past, resolved or occult infection (HBsAg-, HBsAb+/−, HBeAg-, HBeAb+/−, HBcAb-IgM+/−); 5 = Uninfected (HBsAg-, HBsAb-, HBeAg-, HBeAb-, HBcAb-IgM-)

### Hepatitis B infection stages

HBV infection stages are summarized in Table 2. Rates of acute infection (5.7 %) was higher in HIV-1 infected IDUs versus HIV-1 uninfected IDUs (1.4 %), HIV-1 infected non-IDUs (0.0 %), HIV-1 uninfected non-IDUs (0.0 %) and HIV-1 uninfected non-drug users (1.5 %). Likewise, higher rates of chronic infection were detected in the HIV-1 infected IDUs (5.1 %) compared to HIV-1 uninfected IDUs (0.9 %), HIV-1 infected non-IDUs (3.6 %), HIV-1 uninfected non-IDUs (0.0 %) and HIV-1 uninfected non-drug users (1.5 %). In addition, significantly higher rates of vaccine type response was detected in HIV-1 uninfected IDUs (15.4 %) in comparison to HIV-1 infected IDUs (6.4 %), HIV-1 infected non-IDUs (6.5 %), HIV-1 uninfected non-IDUs (10.4 %) and HIV-1 uninfected non-drug users (5.7 %; *P* = 0.003). Moreover, rates of past or resolved infection were non-significantly higher in the HIV uninfected injection drug users (11.2 %) compared to HIV-1 infected IDUs (8.3 %), HIV-1 infected non-IDUs (7.2 %), HIV-1 uninfected non-IDUs (6.3 %) and HIV-1 uninfected non-drug users (6.7 %; *P* = 0.479). Overall, frequencies of susceptible individuals were significantly higher in HIV-1 uninfected non-drug users (84.5 %), HIV-1 infected non-IDUs (82.7 %), and HIV-1 uninfected non-IDUs (83.3 %) compared to HIV-1 infected IDUs (74.5 %) and HIV-1 uninfected IDUs (71.0 %; *P* = 0.005). Thus, HBV exposure rates were 15.5 %, 17.3 %, 16.7 %, 25.5 % and 29.0 %, respectively, in HIV-1 uninfected non-drug users, HIV-1 infected non-IDUs, HIV-1 uninfected non-IDUs, HIV-1 infected IDUs and HIV-1 uninfected IDUs.

### HBV genotypes

**Fig. 1:**
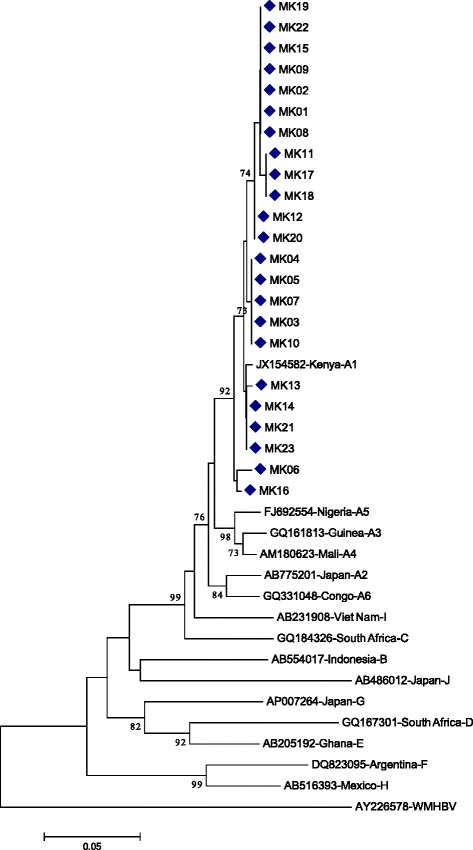
Phylogenetic tree of HBV isolates. Neighbour-Joining method based on 1000 bootstrap replicates and p-distances were used for generating the phylogenetic tree [23]. HBV genotype sequences from GenBank together with their country of origin and accession numbers are presented. Wooly monkey HBV (AY226578-WMHBV) was used as the out-group. Relevant bootstrap values are indicated. HBV isolates from study participants are indicated by diamond signs. The scale bar represents genetic distance

From a total of 33 individuals with acute (*n* = 15) and chronic (*n* = 18) stages, 23 (acute, *n* = 12 and chronic, *n* = 11) were successfully sequenced (Table 2). Only HBV genotype A subgenotype A1 were detected among the study groups. Rates of the A1 genotypes were 6.4 %, 1.9 %, 2.2 % and 3.1 % in HIV-1 infected IDUs, HIV-1 uninfected IDUs, HIV-1 infected non-IDUs, and HIV-1 uninfected non-drug users, respectively. In addition, phylogenetic analysis of the 23 isolates indicated minimal diversity (Fig. 1).

## Discussion

Hepatitis B sero-markers, infection stages and genotypes are integral in selecting, initiating and monitoring response to treatment and in sero- and molecular epidemiologic surveillance [8, 25]. Although HBV exhibits geographic and risk-group clustering of genotypes [18], distribution of HBV genotypes in HIV-1 mono- and co-infected injection and non-injection drug users from Africa is largely unknown. This study examined HBV sero-marker reactivity, infection stages and genotypes among HIV-1 infected and uninfected IDUs and non-IDUs resident at coastal Kenya.

The contrasting sero-positivity rates of HBsAg, HBsAb and HBcAb-IgM in HIV-1 infected and uninfected IDUs and non-IDUs reflect frequent HBV exposure to a “competent” (HIV-1 uninfected IDUs) and “incompetent” (HIV-1 infected IDUs) and less exposure to a “competent” (HIV-1 negative non-IDUs) and “incompetent” (HIV-1 positive non-IDUs) immune system. These findings, are in part, consistent with previous studies illustrating contrasting HBsAg, HBsAb and HBcAb positivity in HIV-1 infected and uninfected IDUs [26]. Most importantly, the results also partly mirror HBsAg positivity rates of 7 % in HIV-1 infected female sex workers from Mombasa (the same region as the current study area) [13]. The lower rates of HBsAg positivity in the HIV-1 negative IDUs and non-drug users, and HIV-1 infected non-IDUs may be linked to perinatal and/or early childhood acquisition of HBV infection [27, 28]. However, the higher rates of HBsAg in the HIV-1 infected IDUs suggest secondary acquisition of HBV post-HIV infection from increased high risk injection and sexual practices that are prevalent among HIV infected IDUs at Coastal Kenya [29]. While the underlying mechanisms remain undefined, it is possible that depleted CD4+ T cell counts in HIV-1 infected IDUs, cause diminished HBsAg sero-conversion and antibody production [30]. HBeAg and HBeAb rates observed in this study are partly consistent with previous studies among HIV-1 positive Nigerian women and Czech IDUs [31, 32]. Altogether, these findings suggest an altered host response to HBV infection in HIV-1 infected and uninfected IDUs and non-IDUs from coastal Kenya.

Although complete serological testing of the five hepatitis B virus sero-markers (HBsAg, HBsAb, HBeAg, HBeAb and HBcAb) is key in guiding accurate diagnosis and infection staging of hepatitis B virus [4, 5], to our knowledge, this is the first study to examine utility of the five hepatitis B virus sero-markers in identifying the infection stages of HBV infections in HIV-1 infected and uninfected IDUs and non-IDUs from Kenya. Although the sensitivity and specificity of various HBV rapid diagnostic tests varies [33, 34], acute hepatitis B infection rates of 5.7 % in the HIV-1 infected IDUs were detected. This finding corroborates with previous studies showing acute hepatitis B virus infection rates of 5.1 % in IDUs from a high HIV-1 risk region of Scotland [35]. In addition, chronic hepatitis B virus infection rates identified in this study are comparable with rates among HIV-1 infected IDUs in the United States of America [36], and female sex workers from Mombasa (a most-at-risk population from the same study area as the current study) [13]. Taken together, the higher rates of acute and chronic hepatitis B virus infection in HIV-1 infected IDUs suggest that HIV-1 infection indirectly and/or directly increase the risk of acquiring and developing hepatitis B virus infection in IDUs. Our laboratory is currently investigating this hypothesis.

Higher rates of vaccine type responses and resolved infection stages in HIV-1 uninfected IDUs are related to the higher CD4+ T cell counts recorded in these study participants. These observations are similar to previous vaccine-type response and resolved infection rates, respectively, detected in young American and Iranian IDUs [37, 38] and HIV-1 uninfected South Africans [39]. The higher CD4+ T cell counts are responsible for increased HBV surface antigen recall responses [40], and resolution of HBV infection [41] in HIV-1 infected and uninfected individuals. Thus, incorporating immune modulators and vaccine booster dose regimens may greatly enhance immunity in co-infected individuals.

Only HBV genotype A sub-genotype A1 were detected in all the study participants, supporting the stability and persistence of this genotype in Kenya. This finding is in line with previous findings in which only genotype A1 was identified among HIV-1 infected adults on antiretroviral therapy from Nairobi [17] and commercial sex-workers from Mombasa [13]. The minimal genetic diversity in the HBV A genotypes found in this study, suggest recent introduction in the HIV-1 infected and uninfected populations of IDUs in Kenya.

Finally, it is important to outline the limitations of this study. This cross-sectional study used HBcAb-IgM to classify acute infection stages even though HBcAb-IgM may persist for several years after acute infection [4]. While the sensitivity and specificity of the various HBV rapid diagnostic tests varies [33, 34], nucleic acid testing was performed on plasma samples obtained from study participants presenting with acute and chronic infection stages based on sero-profile. Even though prospective studies would be important in examining HBV sero-responses, genotypes, viral load and occult infections in HIV-1 infected and uninfected IDUs and non-IDUs, this cross-sectional study has delineated the value of the 5-panel sero-markers in directing infection staging and genotyping of HBV infections.

## Conclusion

HBV 5-panel sero-marker testing is important in guiding infection staging and genotyping of HBV infections. Contrasting sero-reactivities characterized by higher HBsAg and HBcAb positive rates and lower HBsAb positive rates are associated with HIV-1 driven immune suppression. Additionally, it is evident that genotype A is the main circulating HBV infection among IDUs and non-IDUs at coastal Kenya.

## Availability of supporting data

GenBank accession numbers of sequences reported in this study: KP407579, KP407580, KP407581, KP407582, KP407583, KP407584, KP407585, KP407586, KP407587, KP407588, KP407589, KP407590, KP407591, KP407592, KP407593, KP407594, KP407595, KP407596, KP407597, KP407598, KP407599, KP407600 and KP407601.

Phylogenetic data available from the Dryad Digital Repository:http://dx.doi.org/10.5061/dryad.gq8pk
