# Erratum to: Hepatitis B virus sero-profiles and genotypes in HIV-1 infected and uninfected injection and Non-injection drug users from coastal Kenya

**DOI:** 10.1186/s12879-015-1108-4

**Published:** 2015-09-03

**Authors:** Mark K. Webale, Valentine Budambula, Raphael Lihana, Francis O. Musumba, Anthony K. Nyamache, Nancy L. M. Budambula, Aabid A. Ahmed, Collins Ouma, Tom Were

**Affiliations:** Department of Biomedical Sciences and Technology, Maseno University, Maseno, Kenya; Department of Environment and Health Sciences, Technical University of Mombasa, Mombasa, Kenya; Centre for Virus Research, Kenya Medical Research Institute, Nairobi, Kenya; Department of Microbiology, Kenyatta University, Nairobi, Kenya; Bomu Hospital, Mombasa, Kenya; Department of Biological Sciences, Embu University College, Embu, Kenya; African Population and Health Research Centre, Nairobi, Kenya; Department of Medical Laboratory Sciences, Masinde Muliro University of Science and Technology, Kakamega, Kenya

Erratum

After publication of [1] it came to the authors’ attention that Mark K. Webale’s name was displayed incorrectly. The correct spelling of their name has been updated in the original article and is included in this erratum.
